# Prediction of Alzheimer’s disease using blood gene expression data

**DOI:** 10.1038/s41598-020-60595-1

**Published:** 2020-02-26

**Authors:** Taesic Lee, Hyunju Lee

**Affiliations:** 1 Department of Biomedical Science and Engineering, Gwangju Institute of Science and Technology, Gwangju, South Korea; 2 Artificial Intelligence Graduate School, Gwangju Institute of Science and Technology, Gwangju, South Korea; 3 School of Electrical Engineering and Computer Science, Gwangju Institute of Science and Technology, Gwangju, South Korea

**Keywords:** Data mining, Microarrays

## Abstract

Identification of AD (Alzheimer’s disease)-related genes obtained from blood samples is crucial for early AD diagnosis. We used three public datasets, ADNI, AddNeuroMed1 (ANM1), and ANM2, for this study. Five feature selection methods and five classifiers were used to curate AD-related genes and discriminate AD patients, respectively. In the internal validation (five-fold cross-validation within each dataset), the best average values of the area under the curve (AUC) were 0.657, 0.874, and 0.804 for ADNI, ANMI, and ANM2, respectively. In the external validation (training and test sets from different datasets), the best AUCs were 0.697 (training: ADNI to testing: ANM1), 0.764 (ADNI to ANM2), 0.619 (ANM1 to ADNI), 0.79 (ANM1 to ANM2), 0.655 (ANM2 to ADNI), and 0.859 (ANM2 to ANM1), respectively. These results suggest that although the classification performance of ADNI is relatively lower than that of ANM1 and ANM2, classifiers trained using blood gene expression can be used to classify AD for other data sets. In addition, pathway analysis showed that AD-related genes were enriched with inflammation, mitochondria, and Wnt signaling pathways. Our study suggests that blood gene expression data are useful in predicting the AD classification.

## Introduction

Alzheimer’s disease (AD), the most common form of dementia, is estimated to affect in 13.8 million individuals in the United States (US), with 7.0 million being aged 85 years or older by 2050^1^. Based on the National Institute of Neurological, Communicative Disorders, and Stroke and Alzheimer’s Disease and Related Disorders Association (NINCDS-ADRDA) criteria in 1985, probable or possible AD was diagnosed based on subjective symptoms and questionnaires^2^. Recently, the transition from symptom-based to pathophysiology-based AD diagnosis showed that AD diagnosis is mainly based on structural brain changes (MRI), molecular neuroimaging changes (positron emission tomography imaging), and alterations in cerebral spinal fluid biomarkers^3^. Although the elucidation of the biological basis of AD has resulted in many advancements^3^, early diagnostic detection of AD remains challenging.

Recent advances in biotechnology have led to full-scale analyses of the genome, transcriptome, and epigenome rather than focusing on a few biomarkers. A large-scale genome-wide association study (GWAS) of 2,032 individuals with AD and 5,328 controls was presented in 2009 and it identified variants at CLU and CR1, which were associated with AD^4^. Additionally, a meta-analysis of four previously reported GWAS datasets (17,008 AD cases, 37,154 controls) yielded 11 new loci of susceptibility to AD^5^. Recently, Xu *et al*. constructed an AlzData database integrating data from GWAS, eQTL, interactome, and laboratory experiments^6^, which provides all human genes with scores for association with AD, called the Convergent Functional Genomics (CFG) score^7,8^.

In recent years, two large multi-center studies were conducted to identify biomarkers for early AD diagnosis and MCI progression to AD: the Europe-based ANM and US-based Alzheimer’s Disease Neuroimaging Initiative (ADNI)^9,10^. Furthermore, a large amount of publicly available gene expression data on AD have been provided in the NCBI GEO^11^. As a result, various studies, especially gene expression-based studies, have been published to uncover the informative genes associated with AD. The BrainNet study analyzed 113 samples of well-characterized postmortem brain tissues, yielding 21 genes dysregulated in AD cases^12^. A study by Liang *et al*.^13^ consisting of 87 brain tissues samples revealed that in brain tissues of AD cases, the genes encoding subunits of the mitochondrial components showed significantly lower expression. By analyzing RNA expression from brain tissues of AD patients, Xu *et al*.^6^ demonstrated that an early alteration of YAP1 could promote AD.

Although several studies involving gene expression data have uncovered valuable patterns, most gene expression data were obtained from biopsy or autopsy-based samples, which are difficult to extrapolate to clinical settings. Only a few studies used blood-based expression data for uncovering key genes related to AD or predicting early AD^14^. Cooper *et al*.^14^ published a study consisting of 186 AD cases, 118 MCI cases, and 204 controls from three independent datasets, overall suggesting that progranulin expression levels in the blood are increased in AD and MCI.

In Table 1, we list previously published blood expression-based studies for the identification of patients with AD, especially focusing on machine learning (ML)-based studies^15–20^. Detailed information about related works is presented in Supplementary File. The difference between the studies outlined in Table 1 and ours are as follows. First, other studies selected AD-related genes only using statistical or ML methods. We extracted AD-related genes not only via statistical methods, but also protein-protein interaction databases (DBs), transcription factor (TF) DBs, and the CFG method that integrated results from SNP, transcripts, AD-animal model, and text-mining. Besides, we validated selected genes by measuring predictive performances among different datasets. Although studies discriminating AD by blood-based transcriptomic data have been performed, it is unclear whether the classifiers trained using one AD gene expression data set can be applied to other AD data sets. Thus, here, we systematically evaluated five feature selection methods and classifiers for distinguishing individuals with AD from healthy controls (CNs) using three independent blood gene expression datasets. Lastly, we analyzed the biological functions of AD-related genes from the blood via pathway analysis, and compared the result from blood bio-signature with those from brain bio-signature.

**Table 1:** Summary of six studies for predicting AD using blood gene expression data.

Study	Data source/# of AD and CN (Training and test datasets)	Feature selection methods (Data used for feature selection)	Classifying method	Number of selected features	Performance
Booij *et al*.^15^	Not publicly available (Norway)/126 AD and 126 CN (Randomly dividing all data into training and test datasets by 3:1 ratio)	Jack-knife (training data)	PLSR	1239 genes	ACC: 0.87 AUC: 0.94
Lunnon *et al*.^16^	ANM/104 AD and 104 CN (Randomly dividing AD and CN data into training and test datasets by 3:1 ratio)	t-test RF with Meng score and backward elimination (training data)	RF	50 probes	ACC: 0.75
Sood *et al*.^17^	ANM1 and ANM2/49 AD and 64 CN, 40 AD and 71 CN (LOOCV)	Bayesian statistic (ULSAM Ageing data GEO:GSE60862)	kNN	150 probes	AUC: 0.73 (ANM1) AUC: 0.66 (ANM2)
Voyle *et al*.^18^	ANM1 and ANM+DCR/100 AD and 107 CN, 118 AD and 118 CN (ANM1 for training, ANM2 + DCR for test)	REF and pickSizeTolerance (Training data)	RF	13 probes (12 genes)	ACC: 0.657 AUC: 0.724
Li *et al*.^19^	ANM1 and ANM2/145 AD and 104 CN, 140 AD and 135 CN (ANM1 for training, ANM2 for test and vice versa)	Ref-REO (Training data)	Not described	1,145 gene pairs (ANM1: training data) 1,249 gene pairs (ANM2: training data)	AUC: 0.733 (ANM2: test set) AUC: 0.775 (ANM1: test set)
Li *et al*.^20^	ANM1 and ANM2/143 AD and 104 CN, 102 AD and 78 CN (ANM1 for training, ANM2 for test and vice versa)	LASSO regression (ANM1 and ANM2)	Majority voting of SVM, RR and RF	6 genes (Full6set)	AUC: 0.866 (ANM2: test set) AUC: 0.864 (ANM1: test set)

AD: Alzheimer’s Disease; CN: healthy control; PLSR: partial least square regression; ACC: accuracy; AUC: area under the curve; ANM: AddNueroMed; RF: Random Forest; kNN: k-nearest neighbors; RFE: recursive feature elimination; pickSizeTolerance: a function in caret package^29^; ULSAM: the Uppsala Longitudinal Study of Adult Men; LOOCV: leave-one-out cross-validation; LASSO: least absolute shrinkage and selection operator; SVM: support vector machine; RF: random forest; RR: logistic ridge regression.

## Methods

The ADNI consisted of participants recruited at 57 sites in the US and Canada, funded as a private-public partnership^9^. The ANM consortium is a large cross-European AD biomarker study and a follow-on DCR cohort in London^10^. In both ADNI and ANM, AD was diagnosed using the NINCDS-ADRDA criteria for possible or probable AD^2^.

**Figure 1:**
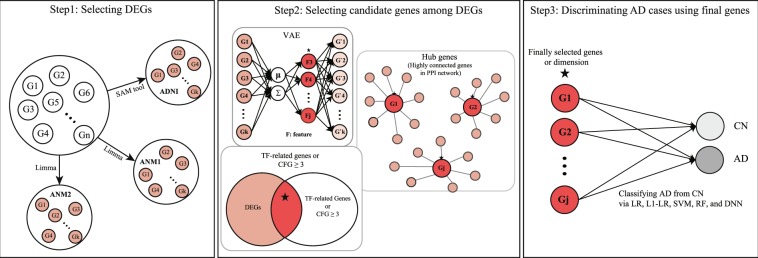
A framework of the study. The informative genes were selected using a training set by two processes: (Step 1) Extracting DEGs; (Step 2) Selecting informative genes using feature selection methods, including VAE, TF-related genes, hub genes, and the CFG scoring; (Step 3) Learning a prediction model using a training set, and predicting a test set by employing five classification methods, including logistic regression (LR), L1-regularized LR (L1-LR), SVM, RF, and DNN. Stars denote the best performance among five classifying methods. DEG, differentially expressed gene; SAM, significance analysis of microarray; ADNI, Alzheimer’s Disease Neuroimaging Initiative; ANM, AddNeuroMed; VAE, variational autoencoder; TF, transcription factor; PPI, protein-protein interaction; CFG, Convergent Functional Genomics; LR, logistic regression; SVM, support vector machine; RF, random forest; DNN, deep neural network; CN, healthy control; AD, Alzheimer’s disease.

We employed three large-scale blood gene expression datasets: ANM1 (GEO:GSE63060), ANM2 (GEO:GSE63061), and ADNI (adni.loni.usc.edu, last downloaded 2018/8/31). The overall framework of our study is illustrated in Fig. 1. The performance was evaluated via internal validation (five-fold CV within each dataset) and external validation (training and test sets from different datasets). In the internal validation. Detailed information about performance assessment is described in the Supplementary File.

The extraction of differentially expressed genes (DEGs), logistic regression (LR), L1-LR, support vector machine (SVM), and random forest (RF) were implemented using R (version 3.4.0) and Bioconductor (release 3.8)^21,22^. Variational autoencoder (VAE) and deep neural network (DNN) were implemented with C++ based TensorFlow with a Python interface^23^.

### Preprocessing of data

Gene expression data were produced using the Affymetrix Human Genome U 219 array for the first case-control study (ADNI), Illumina Human HT-12 v.3 Expression BeadChips for the second case-control study (ANM1), and Illumina Human HT-12 v4 Expression BeadChips for the third case-control study (ANM2).

We performed three steps of data processing. The first step involved the selection of samples and probes to be analyzed. For ADNI, we selected high-quality RNA samples with RIN ≥ 6.9, as the previous study performed^24^, and for ANM1 and ANM2, we did not exclude any samples. The ADNI, ANM1, and ANM2 datasets consisted of 49,386, 30,063, and 29,485 probes, respectively. The median RNA expression value of ADNI, ANM1, and ANM2 were 3.897, 7.584, and 6.154, respectively, indicating that ADNI could be influenced by background noise due to relatively low gene expression intensities. To reduce the background noise of ANDI, we excluded probes with an intensity value ≤ the median of all gene expression values in 100 or more samples, as performed in the previous study^25^. If there were multiple probes annotated in one gene, then the median value of those was selected, yielding 11,276, 21,698, and 22,338 unique probes in ADNI, ANM1, and ANM2, respectively. We selected only probes that were present in all three datasets, and 8,835 final probes were left for analysis.

The second step was normalization within each dataset and renormalization between datasets. The probe set level intensities of all three datasets were normalized by the Robust Multi-Array Analysis (RMA) method^26^. Although each dataset was normalized, a variance or batch effect among different datasets remained. Therefore, we renormalized all three datasets to reduce the batch effect among different datasets using ComBat from the sva package in R^27^.

The third step was selecting DEGs of patients with AD. We extracted DEGs between the control and AD in the ADNI by the significance analysis of microarrays (SAM), which is a method for identifying genes on a microarray with statistically significant changes in expression^28^. It was developed in the context of an actual biological experiment. DEGs of AD in ANM1 and ANM2 were curated via “lmFit” and “eBayes” functions in the limma package, which is based on a linear regression method^29^. We set the cutoffs of FDR of the SAM and limma to 0.05 and 0.01, respectively.

### Feature selection

We used VAE to extract a representation from a set of input features thereby reducing the dimensions of the data^30^. An autoencoder is a type of neural network used to learn efficient and representative information in an unsupervised manner. Specifically, VAE not only adopts the autoencoder architecture but also assumes that the distribution of encoding features is similar to that of original features^30^. The framework of VAE is concisely described in Fig. 1 and precisely in Supplementary Fig. S1A. The structure of VAE is described in Supplementary File.

We obtained information on TF-related genes from the TRANSFAC database 7.0, which is publicly available at http://gene-regulation.com ^31^. We selected “Factor”, “homo sapiens”, and “Organism Species (OS)” as the values of “option”, “search term”, and “table field to search in”, respectively, yielding a list of 608 TF-related genes. Then, common genes between the TF-related genes and DEGs were used as input features of a classifier. Detailed information about the 608 genes and the TF-related genes overlapped with DEGs is presented in Supplementary Table S1.

To curate hub genes, we obtained gene-gene interaction (GGI) data, including 7,765 genes and 54,719 interactions from the Human Protein Reference Database, publicly available at http://www.hprd.org/ ^32^. The process of extracting hub genes among DEGs consisted of several steps. We mapped DEGs obtained from each training set onto the data of GGI and calculated the number of edges of DEGs. To select a specific number of edges, we applied 10 different numbers of edges as thresholds ranging from 10 to 20 to ADNI, ANM1, and ANM2. Among the 10 thresholds, we selected a value of 10, which was similar to the number of CFG-based genes. Therefore, we defined genes with more than 10 edges as hub genes, which interact with more than 10 genes in the GGI database.

The CFG approach is a translational methodology that integrates multiple lines of external evidence from human and animal model studies^7,8^. There have been several studies using various CFG scoring methods, from which we selected two representative methods^6,33^. The first method^6^ was to score genes validated by multi-genomic and experimental studies using the five criteria (Supplementary File). We assigned each DEG one point if the DEG satisfied one of the above five criteria, yielding a score ranging from 0 to 5 points by using a publicly accessible database at http://alzdata.org ^6^. The second method is the database-based CFG scoring method that uses external lines of evidence^33^. We scored a gene as one point if the gene was included in the AD-related genes extracted from two databases, including Alzgene and DigSee^34,35^, yielding a score ranging from 0 to 2. Bertram *et al*. constructed a publicly available, continuously updated database (AlzGene, http://www.alzgene.org) by performing systematic meta-analysis for each polymorphism with available genotype data in at least three case-control subjects^34^. The DigSee extracted gene-disease relationships by incorporating the text-mining method and the ML technique and included 4,494 disease types and 13,054 genes^35^. From the AlzGene and DigSee, we obtained a list of 680 and 1602 AD-related genes, respectively. Combining these two CFG scoring methods, we annotated all DEGs with numeric points ranging from 0 to 7. We defined DEGs with 3 or more points as highly informative AD genes. Lists of DEGs with CFG score (ADNI, ANM1, and ANM2) are presented in Supplementary Tables S2–S4.

### Classifying methods

We utilized LR, L1-GLM, RF, SVM, and DNN as a classifying model. LR, developed by David Cox in 1958, is a standard method for binary classification^36^. L1-LR, first suggested by Tibshirani in 1996, is an extended version of LR applied by LASSO^37^. Due to a sparsity, the L1-LR can simultaneously perform two tasks: feature selection and classification^38,39^. The L1-LR needs λ, the tuning hyperparameter that controls the degree of the penalty, which is typically set as a value that gives the best performance via CV. However, the previous study showed that most weights were penalized to 0 after selecting λ by CV^20^. Therefore, we preliminarily applied 100 sequencing λ values ranging from 10^−3^ to 10^−5^. The larger lambda was selected, the smaller number of genes were selected. Of 100 λ values, we selected the 50^th^ λ value (λ = 0.0001) because this value is the elbow point, where the increasing tendency of the number of selected genes is reduced (Supplementary Fig. S2).

SVM, a robust ML method, is typically used to solve binary classification problems by finding a hyper-plane that maximizes the margin between two classes. Two hyperparameters are needed for the SVM algorithm: cost (C), which indicates the degree of penalty for misclassification, and gamma (γ), which defines the extent of the influence of a single training example. In this study, we adopted the Gaussian radial basis kernel for SVM. The “svm” function in the e1071 package was used to run the SVM algorithm, in which, by default, C and γ values were set to 1 and 1/(dimension of input features)^40^, respectively. Note that another widely used package, Scikit-learn^41^, also adopts default values of C and γ, similar to those of the e1071 package.

The RF algorithm, developed by Leo Breiman, utilizes an ensemble of classification trees, which include bootstrap samples and randomly selected variables^42,43^. Two hyperparameters are required in RF: the number of trees, ntree, and the number of randomly selected features, mtry. In this study, we determined ntree = 500, and ntry = $\sqrt{{\rm{data}}\,{\rm{dimension}}}$, which were default values.

DNN is a method of learning representative features with multi levels of representation, obtained via non-linear perceptrons, each of which transforms the representation at one level to that at a higher or abstract level with reduced dimension^44,45^. LeCun *et al*.^44^ suggested that these higher levels of representation would amplify important aspects of the input for classification tasks and suppress irrelevant variations. In this study, a DNN architecture consists of two hidden layers, where the number of hidden nodes are $[\frac{{\rm{N}}}{2}]$ and $[\frac{{\rm{N}}}{4}]$ (N: number of input features) in the first and second hidden layers, respectively. We set the minimum of hidden nodes in the first and second hidden layers as 10 and 5, respectively. The hyperbolic tangent function between hidden layers and the sigmoid function in the final layer were employed. We hypothesized that more input features needed more iterations of training. Therefore, we determined the number of iteration as “N (number of input features) × 3”, and also set the minimum and maximum number of iteration as 200 and 3000, respectively. We optimized our DNN model utilizing the Adagrad optimizer^46^ when an input dimension was ≥ 800 or the AdamOptimizer when an input dimension was <800^47^, and a learning rate was set to 0.001.

## Results

### Internal validation (CV within each dataset)

For samples in ADNI, ANM1, and ANM2, the average chronological ages were 75.9, 74.1, and 76.7, and ratios of males were 49.7, 35.3, and 39.4%, respectively (Table 2). In ADNI, ages did not differ significantly between AD and CN samples, while ages in ANM1 and ANM2 showed a significant difference between AD and CN samples. The gender difference between AD and CN samples was not significant in all datasets (Table 2).

**Table 2:** Demographic characteristics in ADNI, ANM1, and ANM2.

	ADNI			ANM1			ANM2		
	CN	AD	P value	CN	AD	P value	CN	AD	P value
Number of samples, n	136	63		104	145		134	139	
Age, years	75.62 ± 6.76	76.51 ± 7.63	0.43	72.38 ± 6.34	75.40 ± 6.58	<0.001	75.29 ± 6.02	77.89 ± 6.67	<0.001
Gender (male), n	64 (47.1)	35 (55.6)	0.265	42 (40.4)	46 (31.7)	0.159	53 (39.6)	54 (38.8)	0.762

ADNI: Alzheimer’s Disease Neuroimaging Initiative; ANM: AddNeuroMed; CN: healthy control; AD: Alzheimer’s disease; Continuous variables are presented as mean ± standard deviation, and categorical variables are as number (percent, %).

**Figure 2:**
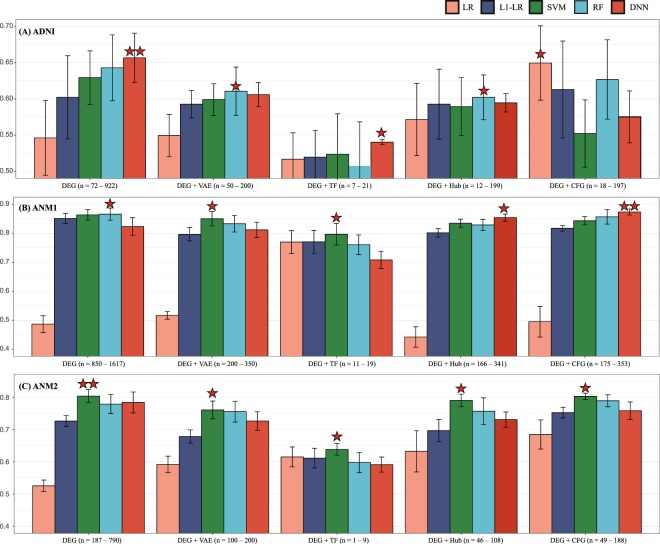
Internal validation. (**A**–**C**) illustrate performance (y-axis: AUC) of classifying AD from CN in ADNI, ANM1, and ANM2, respectively. X-axes of (**A**–**C**) describe feature selection methods, including DEG, VAE, TF, and CFG. Different colors of columns indicate classifiers, including LR, L1-LR, SVM, and DNN. One star denotes a model with the best performance among five classifiers, and two stars denote the best model among five classifiers and five feature selection methods. The ranges of numbers indicate the numbers of selected genes in each feature selection method, and all numbers of selected genes are described in the Supplementary Table S5. AUC, area under the curve; AD, Alzheimer’s disease; ADNI, Alzheimer’s Disease Neuroimaging Initiative; ANM, AddNeuroMed; DEG, differentially expressed gene; VAE, variational autoencoder; TF, transcription factor; LR, logistic regression; L1-LR, L1-regularized LR; SVM, support vector machine; RF, random forest; DNN, deep neural network.

In the internal validation (five-fold CV, ADNI), DEGs ranging from 72 to 922 were curated from each training set, and are presented in Supplementary Table S5. Similarly, DEGs ranging from 850 to 1617 and those from 187 to 790 were selected in each five-fold CV for ANM1 and ANM2, respectively (Supplementary Table S5). The numbers of DEGs of ADNI varied most with a standard deviation (SD) of 352.7 while those of ANM2 were most consistent (an SD of 254.4). When all DEGs were used as input features in ADNI, DNN and RF outperformed the other methods (Fig. 2A). In ANM1, L1-LR, SVM, RF, and DNN showed AUC values greater than 0.80 (Fig. 2B). In ANM2, SVM was the best performing classifier for distinguishing AD when all DEGs were used as input features (Fig. 2C).

In ADNI, ANM1 and ANM2, when the VAE method was used for dimension reduction, the performances were improved only for the LR classifier compared with the DEGs. For the other methods, the application of VAE may lose important information (Fig. 2, Supplementary Table S5).

We extracted TF-related genes ranging from zero to 21 among DEGs of ADNI (Supplementary Table S5). In the same way, 11 to 19 TF-related genes and zero to 9 TF-related genes were selected for ANM1 and ANM2, respectively. In ADNI, ANM1, and ANM2, the mean AUC values of all classifying methods, except for LR, decreased when a set of TF-related genes were used as input features (Fig. 2).

We selected 12 to 199, 166 to 341, and 46 to 108 hub genes with more than 10 edges in the GGI network among DEGs in ADNI, ANM1, and ANM2, respectively (Supplementary Table S5). As a result, the majority of classifying methods did not show improved performance, except for LR, DNN, and LR in ADNI, ANM1, and ANM2, respectively (Fig. 2).

The CFG method was utilized to select 18 to 197 genes from DEGs in ADNI, showing that LR and L1-LR showed improved performance. In ANM1 and ANM2, after the CFG scoring was used, 850 to 1617 and 187 to 790 of DEGs were shrunk to 175 to 353 and 49 to 188 genes, respectively. Two classifying methods (LR and DNN) in ANM1 and three (LR, L1-LR, and RF) in ANM2 were better than those using all DEGs as input, respectively (Fig. 2).

For each feature selection method, most comparisons across the five classifiers did not yield significant differences in performance (measured by a paired *t*-test, Supplementary File) in ADNI, ANM1, and ANM2 (Supplementary Table S5), except that LR showed statistically lower performances compared to other methods in ANM1 and ANM2.

Among five feature selection approaches, on average, the DEG provided the best performance in ADNI (p = 0.109 measured by a *t*-test, please refer to the Supplementary File) and ANM1 (p = 0.631), and the “DEG + CFG” (p = 0.002) showed the best performance in ANM2 (Fig. 2).

### External validation (Cross datasets analysis)

When 334 DEGs extracted from ADNI served as input features for predicting AD patients in ANM1, the best and second-best performing classifiers were L1-LR and SVM with 0.70 and 0.66 AUC values, respectively. When ADNI and ANM2 were used as training and test datasets with input features of the 334 DEGs, respectively, L1-LR manifested the best AUC (0.69) among classifiers. Note that the process of extracting 334 DEGs in ADNI was completely independent of ANM1 and ANM2 datasets.

**Figure 3:**
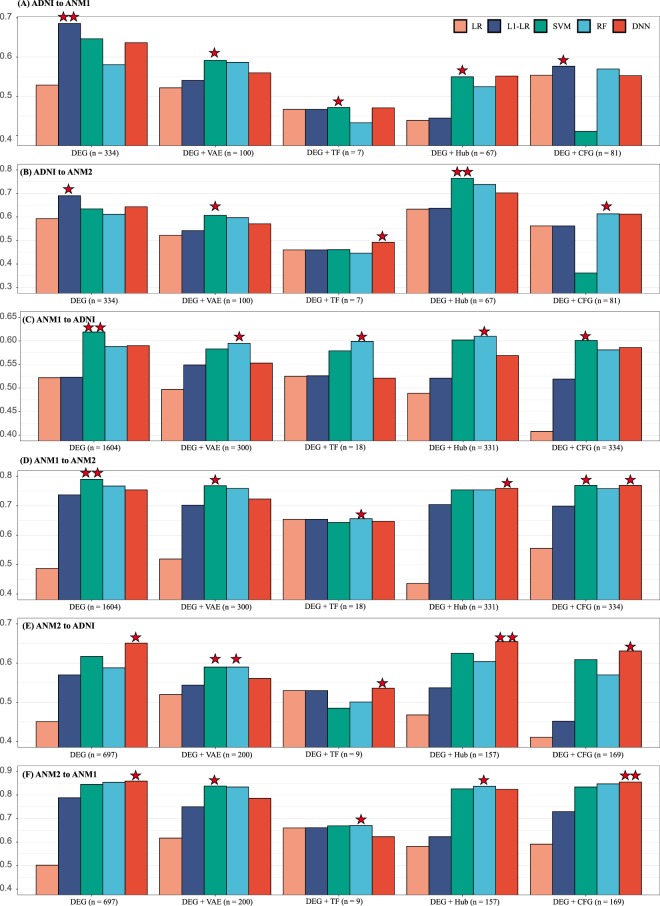
External validation. (**A**) ADNI and ANM1 (**B**) ADNI and ANM2 (**C**) ANM1 and ADNI (**D**) ANM1 and ANM2 (**E**) ANM2 and ADNI (**F**) ANM2 and ANM1 are arranged as training and testing dataset, respectively. The y-axes in each graph show performance measured by AUC. One star denotes a model with the best performance among five classifiers, and two stars denote the best model among five classifiers and five feature selection methods. The number indicates the number of selected genes in each feature selection method. AUC, area under the curve; AD, Alzheimer’s disease; ADNI, Alzheimer’s Disease Neuroimaging Initiative; ANM, AddNeuroMed.

When using VAE (334 to 100) and TF-related genes (seven genes), the performances were not better than that of the method using 334 DEGs were proposed when arranging ADNI and ANM (ANM1, ANM2) as training and testing datasets, respectively (Fig. 3A,B, Supplementary Table S6).

We tested the ANM1 dataset with 67 hub genes selected from ADNI and achieved lower AUC than DEGs. However, the best performance (AUC: 0.76) was acquired when testing ANM2 with the 67 hub genes from ADNI and SVM (Fig. 3B). Using 81 DEGs with CFG ≥ 3 as input features, most classifiers did not show improved performances, except for LR (ANM1) and RF (ANM2) (Fig. 3A,B).

The best performance in ANM1 (testing set 1) was an AUC of 0.70 when the 334 DEGs and L1-LR served as the feature selection method and classifying model, respectively (Fig. 3A). In the case of ANM2 (testing set 2), an AUC of 0.76 was the best result achieved using the hub method and SVM (Fig. 3B).

When 1604 DEGs from ANM1 were used for training, SVM showed the best performance with AUCs of 0.62 and 0.79 for test sets ADNI and ANM2, respectively (Fig. 3C,D). When we used VAE (1604 to 300), 18 TF-related genes, 331 hub genes, and the CFG method, performances of most classifying methods decreased. The best performances in ADNI and ANM2 occurred when DEGs and SVM were adopted as the input features and the classifier, respectively.

When 697 DEGs from ANM2 were used for training, DNN showed the best performances of 0.65 and 0.85 for both ADNI and ANM1, respectively (Fig. 3E,F). Using reduced 200 dimensions from 697 DEGs via VAE and TF-related genes as input, overall performance decreased compared with using 697 DEGs. When testing ADNI, the hub gene offered the best performance (AUC: 0.66, DNN); however, the CFG performed best when testing ANM2 (AUC: 0.86, DNN).

In the comparisons across the five classifiers, the performances of four classifiers (L1-LR, SVM, RF, and DNN) were enhanced as compared with that of LR. Among the four classifiers, most comparative analyses showed an insignificant difference in terms of performances (*p*-values were measured by Venkatraman’s method, refer to the Supplementary File and Supplementary Table S6).

Among five feature selection methods, on average, the DEG (p = 0.146 measured by a *t*-test, Supplementary File) provided the best performance compared to other methods when ADNI and ANM1 were arranged as training- and test-sets, the “DEG + Hub genes” (*t*-test, p = 0.2) showed the best performance between ADNI (training) and ANM2 (test), and the “DEG + CFG” (*t*-test, p = 0.039) yielded the best result between ANM1 (training) and ANM2 (test).

### Pathway analysis of informative genes in each dataset

We found that the performances of discriminating AD were best when “DEG” or “DEG + CFG” were used as feature selection methods. Therefore, we performed pathway analysis of DEGs (334 genes of ADNI, 1604 of ANM1, and 697 of ANM2) and “DEG + CFG” (81 genes of ADNI, 334 of ANM1, and 169 of ANM2) using the KEGG pathways^48^ and Gene Ontology^49^ obtained from Molecular Signature Database (MSigDB)^50^. We removed general pathways that consist of ≥ 500 genes. A pathway enrichment test was performed using a hypergeometric test followed by multiple comparison correction (Benjamini–Hochberg method) and pathways with q-values <0.05 were considered significantly enriched. As a result, no pathways (ADNI: DEG), 26 (ANM1: DEG), 57 (ANM2: DEG), 7 (ADNI: DEG + CFG), 340 (ANM1: DEG + CFG), and 366 pathways (ANM2: DEG + CFG) were selected (Supplementary Table S7).

**Figure 4:**
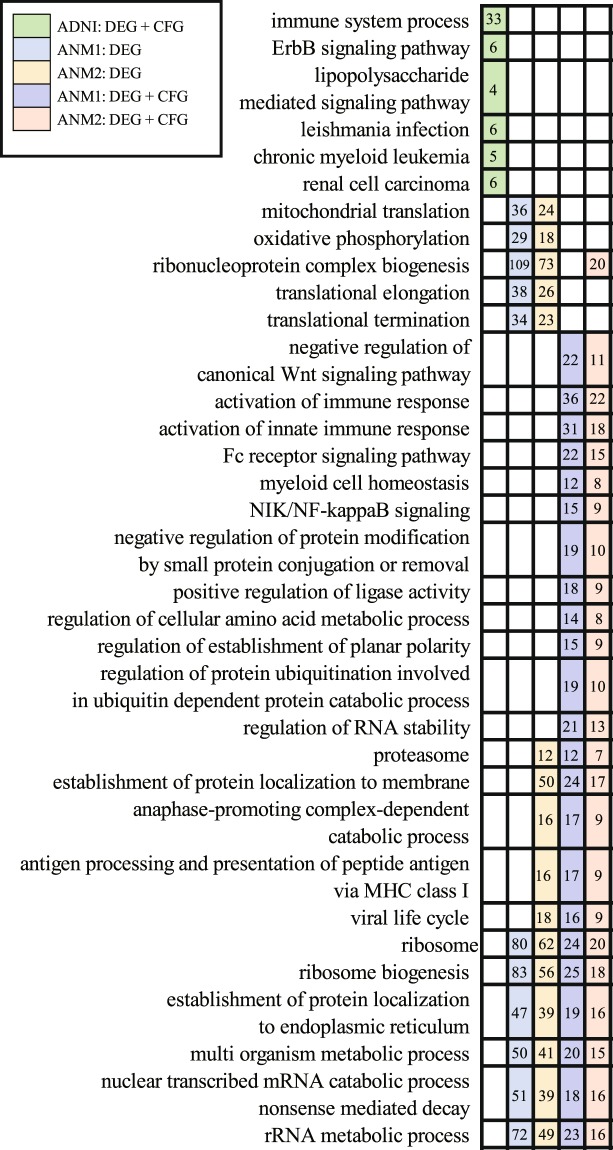
Significant pathways in ADNI, ANM1, and ANM2. Right-side matrix is marked with the color assigned to each data if the genes of each data are enriched in left-side pathways. Numbers in each square indicate selected genes among different pathways. ADNI, Alzheimer’s Disease Neuroimaging Initiative; ANM, AddNeuroMed.

In ADNI, the representative pathways enriched via 81 genes (DEG + CFG) were as follows: immune system process, ErbB signaling, and lipopolysaccharide mediated signaling pathways (Fig. 4).

When using only DEG in ANM1 and ANM2, 26 common pathways were obtained, and the following pathways were notable: mitochondrial translation, oxidative phosphorylation, and ribonucleoprotein complex biogenesis (Fig. 4). DEGs with CFG in ANM1 and ANM2 were commonly enriched in 51 pathways, such as negative regulation of canonical Wnt signaling pathway, activation of the immune response, and myeloid cell homeostasis (Fig. 4).

Common pathways among four lists of pathways (two feature selection methods and two datasets [ANM1, ANM2]) were 17, and representative pathways are as follows: ribosome, the establishment of protein localization to the endoplasmic reticulum, and multi organism metabolic process (Fig. 4). Collectively, highly informative genes in ADNI were enriched in immune and inflammatory pathways. Differentially expressed genes from ANM were associated with energy metabolism (mitochondria and oxidative phosphorylation), and “DEG + CFG” from ANM showed significant enrichment for Wnt-related and immune pathways (Fig. 4).

### Common bio-signature between blood and brain AD samples

**Figure 5:**
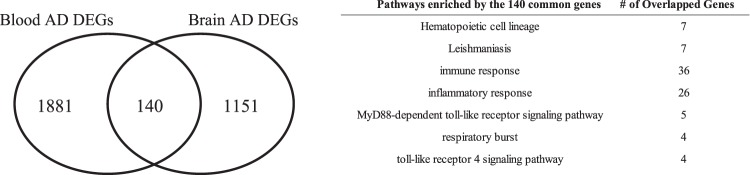
Common AD bio-signatures between blood and brain. AD, Alzheimer’s Disease; DEG, differentially expressed gene.

We analyzed the brain gene expression datasets (GEO: GSE33000), including 157 AD samples and 310 CN. We curated 1291 DEGs using the “lmFit” function in the limma package followed by a multiple comparison correction and a fold change (FC) (FDR <0.05 and | log_2_(FC)| > 0.2). When comparing 2021 blood DEGs (union of DEGs among ADNI, ANM1, and ANM2), 140 of 1291 brain DEGs were common with blood DEGs, which were enriched with two KEGG and 31 GO pathways (Fig. 5). Representative pathways included immune response, inflammatory response, MyD88-dependent toll-like receptor signaling, and toll-like receptor 4 signaling pathways (Fig. 5).

## Discussion

In this study, we identified AD-related genes by means of DEGs, the TF database, connectivity in the gene network, and CFG. Considering both internal and external validations, this study showed that all DEGs and the DEGs with CFG could accurately identify AD. Among the previous studies listed in Table 1, a study by Li *et al*.^20^ classified AD patients most accurately in the ANM dataset. However, in their study^20^, it seems that both ANM1 and ANM2 datasets were used for selecting features (six genes). After curating the six AD-related genes, they used them as input features for the SVM model, yielding AUCs of 0.86 and 0.873 when testing ANM1 and ANM2, respectively^20^. With the same six genes and the same training- and test-set described by Li *et al*.^20^, we measured prediction performances and obtained results that were consistent with those of Li *et al*.^20^. Afterward, we predicted an independent data ADNI using these six genes as input features to the SVM method, and obtained AUCs 0.62 (training dataset: ANM1) and 0.57 (ANM2). This method^20^ was not as effective as our method using DEGs as input features to the same method, which had AUCs of 0.62 (training dataset: ANM1) and 0.63 (ANM2).

The primary objective of this study was to investigate the prediction of AD patients using AD-related genes obtained across different datasets. The performances in the external validation were high between ANM1 and ANM2, consistent with the previous studies^18–20^. However, the prediction accuracies were low between ADNI and ANM (ANM1 and ANM2), and there was no study using these datasets as external validation. Several studies have suggested the limitations of ADNI. First, gene expression differences between AD and CN in ADNI were low compared to those in ANM, yielding difficulty in extracting DEGs in ADNI. Li *et al*.^20^ faced a similar problem, and selected DEGs based on nominal *p*-values < 0.05 because no gene passed a multiple testing correction. To overcome this limitation, we removed probes with low intensity by a previously validated method^25^. Furthermore, we attempted several methods to curate DEGs, including a *t*-test, limma^29^, and SAM^28^, and found that SAM, which is based on the permutation, could extract DEGs (FDR < 0.05). Second, we observed that for ADNI, the numbers of DEGs varied most across five CV sets among ADNI, ANM1, and ANM2 (the highest SDs for the numbers of DEGs were as follows, ADNI: 352.7, ANM1: 309.3, ANM2: 254.4). This observation might partially explain why in the internal validation, the AUCs for ADNI were lower than those for ANM1 and ANM2. Third, the qualities of gene expression data for some samples in ADNI were low. When all samples in ANDI were used, performances in both internal and external validations were low. The AUC values of the highest performance classifiers for internal and external validation were only 0.613 (Internal validation, ADNI), 0.601 (External validation, ANM1 to ADNI), and 0.63 (External validation, ANM2 to ADNI), respectively. Thus, in this study, we used gene expression samples with RIN values ≥ 6.9, resulting in increased performances in Figs. 2 and 3.

In the external validation, L1-LR, SVM, and DNN showed the best performance. In a previous study, L1-LR and SVM performed well as a feature selection method and a classifier for AD classification, respectively^20^. We observed that DNN did not outperform other classifiers in all cases, which might be because the relatively small size of samples was composed of high dimensional information (i.e., the number of genes) and the number of samples was insufficient to learn all perceptrons of DNN. Furthermore, when we applied several settings of DNN to improve performance, including drop-out and early stopping, we found that although the maximum AUC value increased, the range of the AUC values become more varied than that of the default setting. In future work, the performance will be improved by adjusting parameters and modifying the architecture of DNN when more data are available^51,52^.

We investigated DEG genes with high CFG scores for their correlation to AD. Among 334 DEGs in the ADNI dataset, four genes (TGFB1, RAB11A, MAPK3, and RTN4) showed a maximum CFG score of five points. TGFB1 reportedly increases the risk of developing late-onset AD^53^, ERK (MAPK3) is expectedly activated in AD brains and involved in tau phosphorylation and amyloid deposition^54^. According to recent reports, two additional genes (RAB11A, RTN4) are also related to AD^55,56^. Among 1604 DEGs in the ANM1 dataset, five genes (TIMP1, CD14, FADD, CAMK2G, and FCER1G) had the highest CFG score of six points. MMP-9-TIMP1 pathway was known to be stimulated by Abeta 25–35 fragment to eliminate amyloid deposition from AD brains^57^. The lipopolysaccharide (LPS) receptor CD14 also reportedly contributes to neuroinflammation in AD^58^. FCER1G is one of the microglial-specific genes, and the microglial is considered a major causal factor in AD^59^. Among 697 DEGs in the ANM2 dataset, ten genes (DBI, CDK5R1, SORL1, CTNNA1, CTSS, CAPN1, NFKBIA, SERPINA1, CST3, and VIM) had maximum scores of five CFG points. CDK5R1 is known to be closely related to AD onset and progression^60^, SORL1 interacts with the movement of APP and plays a possible role in AD progression^61^, whereas CTNNA1 is critical to the folding and lamination of the cerebral cortex and is involved in AD pathogenesis^62^.

We observed several enriched pathways with AD-related genes in blood samples in ADNI and ANM, which were also enriched in human or mouse brain tissues. The main pathways of ADNI were related to immune response. A study that found conserved genetic signals in mice and human brain tissues reported that genes related to early and late-stage AD were significantly enriched in immune system processes^63^. Additionally, infection-related pathways (lipopolysaccharide mediated signaling pathway) were enriched in ADNI, which was activated in the human brain with AD^64^, and associated with the increased risk of AD^65^. The DEGs in ANM were significantly enriched with mitochondria-related pathways. Liang *et al*.^13^ demonstrated that AD cases had significantly down-regulated expression of the nuclear genes encoding subunits of the mitochondrial electron transport chain in several brain regions. The DEGs with CFG ≥ 3 in ANM were enriched with the Wnt signaling pathway, which is associated with the developmental process of the nervous system, and especially its association with synaptogenesis was validated in a mouse brain model^66^ and the protective role of neurodegeneration in AD rat model^67^.

In Table 2, ANM1 and ANM2 showed a significant difference in terms of age between AD and CN. Although gender difference did not significantly differ between AD and CN, other studies reported gender difference in the AD risk^68^. Thus, we adjusted for age as well as gender for the ADNI, ANM1, and ANM2 datasets. Afterward, we measured AD predictive performances using five feature selection methods and five classifiers. The detailed adjustment procedure is described in the Supplementary File. As a result, we observed that classifying performances did not significantly differ after the adjustment (Supplementary Fig. S3).

There are three subtypes of AD, including preclinical AD^69^, prodromal AD^70^, and AD dementia. The subtypes of AD are diagnosed using the amyloid positron emission tomography (PET) scan. However, the ANM datasets (ANM1 and ANM2) did not include amyloid PET results. While the ADNI dataset had amyloid PET data, samples with gene expressions were not explicitly classified by the amyloid PET image. Thus, instead of analyzing the three subtypes of ADs, we analyzed MCI samples in the ADNI, ANM1, and ANM2 diagnosed by the NINCDS-ADRDA criteria^2^. First, we compared FCs between two pairs of datasets, AD *vs*. CN and MCI *vs*. CN, for each dataset (Supplementary Fig. S4). In detail, we compared log_2_FC for all genes (n = 8,835), DEGs (n = 334, 1604 and 697 for ADNI, ANM1, and ANM2, respectively), and DEG with CFG (n = 81, 334, and 169 for ADNI, ANM1, and ANM2, respectively) using Spearman correlation. As a result, we observed positive correlations between log_2_ (AD/CN) and log_2_ (MCI/CN) in all datasets and several gene sets (all genes, DEG, and DEG with CFG) (Supplementary Fig. S4), suggesting that genes are similarly upregulated or downregulated in AD and MCI.

We further integrated the ADNI, ANM1, and ANM2 datasets, and investigated the AD prediction performance on the integrated dataset (Supplementary File). To integrate the three datasets and select features from it, we applied four different approaches. First, the ComBat method, which was mainly used for internal and external validations, was used to remove batch effects among these datasets^27^, and then DEGs were selected using the “lmFit” function in the limma package. Second, the scaling & quartiling method by Mohammadi-Dehcheshmeh *et al*.^71^ was used to remove batch effects further, and then DEGs were selected using the “lmFit” function. Third, DEGs were computed for each dataset via the moderate *t*-test, and then rankings of *p*-values were used to curate meta-DEGs among three datasets^72^. Fourth, DEGs were computed for each dataset via the moderate *t*-test, and then *p*-values were combined via Fisher’s method^73^. The gene expression values normalized by the ComBat method showed significant correlations with those yielded by the other three approaches (Supplementary Fig. S5). Furthermore, the DEGs curated by the first ComBat approach significantly correlated with those by the other three methods (Supplementary Fig. S6). We evaluated the AD predictive performance by the three-fold CV. When five classifiers, LR, L1-LR, SVM, RF, and DNN, were used for training and test, the ComBat approach obtained AUC values of 0.5, 0.7, 0.8, 0.79, and 0.79, respectively (Supplementary Fig. S7). This result shows that these three datasets can be integrated for classifying AD and CN. Also, the ComBat method outperformed the other three methods in terms of the average AUC values of the five classifiers (Supplementary Fig. S7).

Most studies have used the NINCDS-ADRDA criteria made in 1984^2^ for classifying subjects as AD and CN samples. The NINCDS-ADRDA criteria are symptom- and individual doctor-based diagnosis, and therefore, may yield inconsistencies between different datasets. Using a single nucleotide polymorphism dataset in ADNI, Apostolova *et al*.^74^ curated seven variants using brain amyloidosis as a dependent variable, while only one variant (FERMT2) was found using the AD stage determined by the NINCDS-ADRDA as a dependent variable. In addition, Edmonds *et al*.^75^ suggested that some MCI samples diagnosed by the NINCDS-ADRDA are false positives. In 2007, a revised version of the NINCDS-ADRDA criteria, which more focused on the pathology of AD rather than clinical symptoms, was introduced^3^. In the future, if genomic data composing participants determined by the revised diagnostic criteria of AD are available, more AD-related genes and pathways can be identified.

## Conclusions

In this study, we showed that expression values of AD-related genes obtained from blood samples of ADNI, ANM1 and ANM2 could classify AD and CN. Additionally, we observed that AD-related genes from blood samples were enriched with several pathways including immune, inflammation, energy metabolism, and Wnt signaling, which are consistent with observations from brain tissue-based studies. Collectively, AD-related genes from blood samples contribute to the development of blood-based AD diagnostic and treatment tools.

## Supplementary information

Supplementary File.

Supplementary Table 1.

Supplementary Table 2.

Supplementary Table 3.

Supplementary Table 4.

Supplementary Table 5.

Supplementary Table 6.

Supplementary Table 7.

Supplementary Table 8.

## Data Availability

ADNI and ANM datasets are publicly available (ADNI, http://adni.loni.usc.edu/; ANM, https://www.ncbi.nlm.nih.gov/geo/).
