# Supplementary material for: Prediction of Alzheimer’s disease using blood gene expression data
Source: Sci Rep. 2020 Feb 26;10:3485. doi: 10.1038/s41598-020-60595-1 (PMC7044318; doi:10.1038/s41598-020-60595-1)
Supplement: Supplementary file 1 — Supplementary File. [file 41598_2020_60595_MOESM1_ESM.docx]

**Prediction of Alzheimer's disease using blood gene expression data**

**(Supplementary File)**

Taesic Lee^a^, Hyunju Lee^a,b,c.^ *

^a^ Department of Biomedical Science and Engineering, Gwangju Institute of Science and Technology, Gwangju, South Korea.

^b^ Artificial Intelligence Graduate School, Gwangju Institute of Science and Technology, Gwangju, South Korea.

^c^ School of Electrical Engineering and Computer Science, Gwangju Institute of Science and Technology, Gwangju, South Korea.

***Corresponding author**

Hyunju Lee, PhD

Professor, School of Electrical Engineering and Computer Science

Professor, Artificial Intelligence Graduate School

Gwangju institute of science and technology

123 Chemdangwagi-ro, Gwangju, Korea

Postal zip-code: 61005

Tel: (+82) 62-725-2213; Fax: (+82) 62-715-2204

E-mail: [hyunjulee@gist.ac.kr](mailto:hyunjulee@gist.ac.kr)

**Supplementary Tables**

**Supplementary Table 1:** Transcription factor-related genes

**Supplementary Table 2:** A list of differentially expressed genes with convergent functional genomics score for ADNI

1. The Score means the *T*-statistic value.
2. The Numerator column is the mean difference for each gene.
3. The Denominator means standard deviation for feature plus s0 (Computed exchangeability factor)
4. eQTL: # of variants associated with each gene expression in the AD-related CFG database.
5. GWAS: # of AD-related variants in the AD-related CFG database.
6. PPI: AD-core genes physically interacting with each gene in the AD-related CFG database.
7. Early_DEG: genes with early differential expression in AD mice (AD-related CFG database).
8. Pathology.cor..abeta./ Pathology.cor..tau.: genes correlating with AD pathology in AD mice (P < 0.05) at the expression level
9. CFG: The CFG score from the AD-related CFG database
10. alz_gene: AD-related gene from the AlzGene database.
11. digsee: AD-related gene from the DigSee database.
12. Final_CFG: CFG + alz_gene + digsee

**Supplementary Table 3:** A list of differentially expressed genes with convergent functional genomics score for ANM1

1. The log_ratio means Log2(fold change)
2. The AveExpr means ‼
3. The t_value means the *T*-statistic value.
4. The beta means B-statistic (lods or B) which is the log-odds that that gene is differentially expressed.
5. eQTL: # of variants associated with each gene expression in the AD-related CFG database.
6. GWAS: # of AD-related variants in the AD-related CFG database.
7. PPI: AD-core genes physically interacting with each gene in the AD-related CFG database.
8. Early_DEG: genes with early differential expression in AD mice (AD-related CFG database).
9. Pathology.cor..abeta./ Pathology.cor..tau.: genes correlating with AD pathology in AD mice (P < 0.05) at the expression level
10. CFG: The CFG score from the AD-related CFG database
11. alz_gene: AD-related gene from the AlzGene database.
12. digsee: AD-related gene from the DigSee database.
13. Final_CFG: CFG + alz_gene + digsee

**Supplementary Table 4:** A list of differentially expressed genes with convergent functional genomics score for ANM2

**Supplementary Table 5:** Five-fold cross validation results for ADNI, ANM1, and ANM2

ADNI, Alzheimer's Disease Neuroimaging Initiative; ANM, AddNeuroMed; DEG, differentially expressed gene; VAE, variant autoencoder; TF, transcription factor; CFG, Convergent Functional Genomics; LR, logistic regression; L1-LR, L1-regularized LR; SVM, support vector machine; RF, random forest; DNN, deep neural network; CV, cross-validation set; AUC, area under the curve.

**Supplementary Table 6:** External validation using ADNI, ANM1, and ANM2.

ADNI, Alzheimer's Disease Neuroimaging Initiative; ANM, AddNeuroMed; CN, healthy control; AD, Alzheimer’s disease; DEG, differentially expressed gene; VAE, variant autoencoder; TF, transcription factor; CFG, Convergent Functional Genomics; LR, logistic regression; L1-LR, L1-regularized LR; SVM, support vector machine; RF, random forest; DNN, deep neural network; CV, cross-validation set; AUC, area under the curve.

**Supplementary Table 7:** Enriched Alzheimer's disease-related pathways

Category: KEGG-Kyoto Encyclopedia of Genes and Genomes (KEGG) database, GO-Gene ontology (GO) biological process (bp = Biological Process; cc = Cellular Component; mf = Molecular Function)

ADNI_CFG: differentially expressed gene (DEG) with CFG score > 3 in the ADNI

ANM1: DEG in the ANM1

ANM1_CFG: differentially expressed gene (DEG) with CFG score > 3 in the ANM1

ANM2: DEG in the ANM2

ANM2_CFG: differentially expressed gene (DEG) with CFG score > 3 in the ANM2

**Supplementary Table 8:** Enriched pathways by common DEGs between blood and brain AD datasets.

**Supplementary Figures**

**Supplement Figure 1.** Overall framework of VAE

(A) Structure of VAE. (B) distribution of expression of randomly selected 412 genes. (C) distribution of 150 values shrunken from the 412 genes by VAE. (D) Correlational plot between original expression values and reconstructed values from the 150 features.

VAE, Variational autoencoder


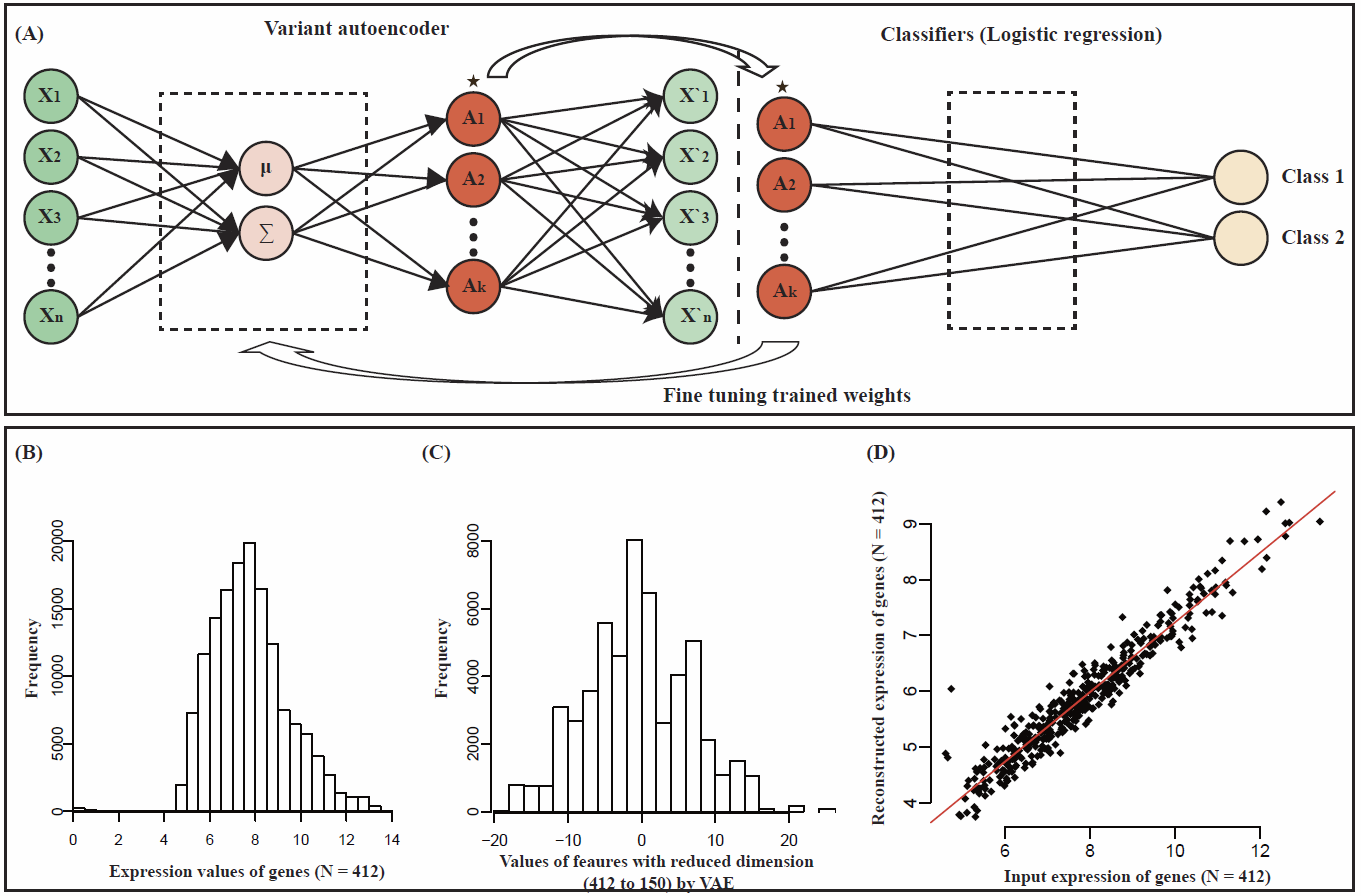


**Supplement Figure 2.** Selected genes in L1-LR as λ. Abbreviations: L1-LR, L1-regularized logistic regression.


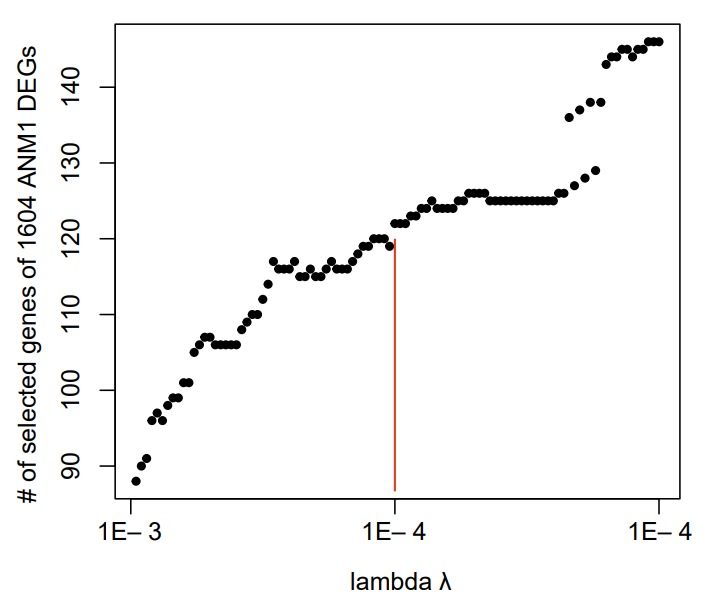


**Supplement Figure 3.** Comparison of classifying performances before and after adjustment of age and gender.


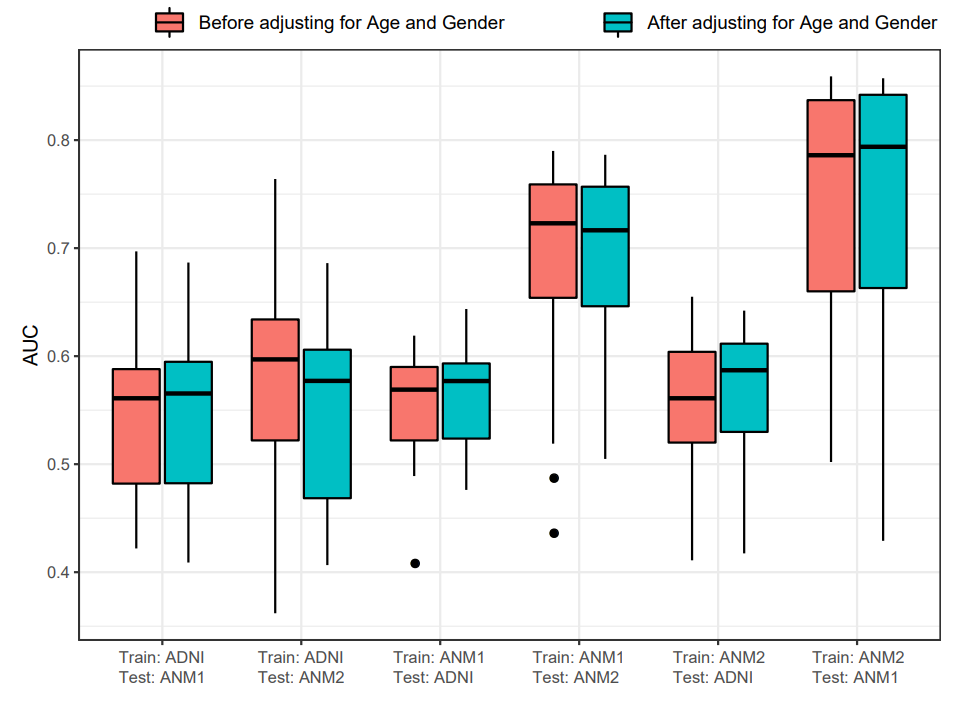


**Supplement Figure 4.** Comparison of MCI and AD subjects.

MCI, Mild cognitive impairment; AD, Alzheimer’s disease


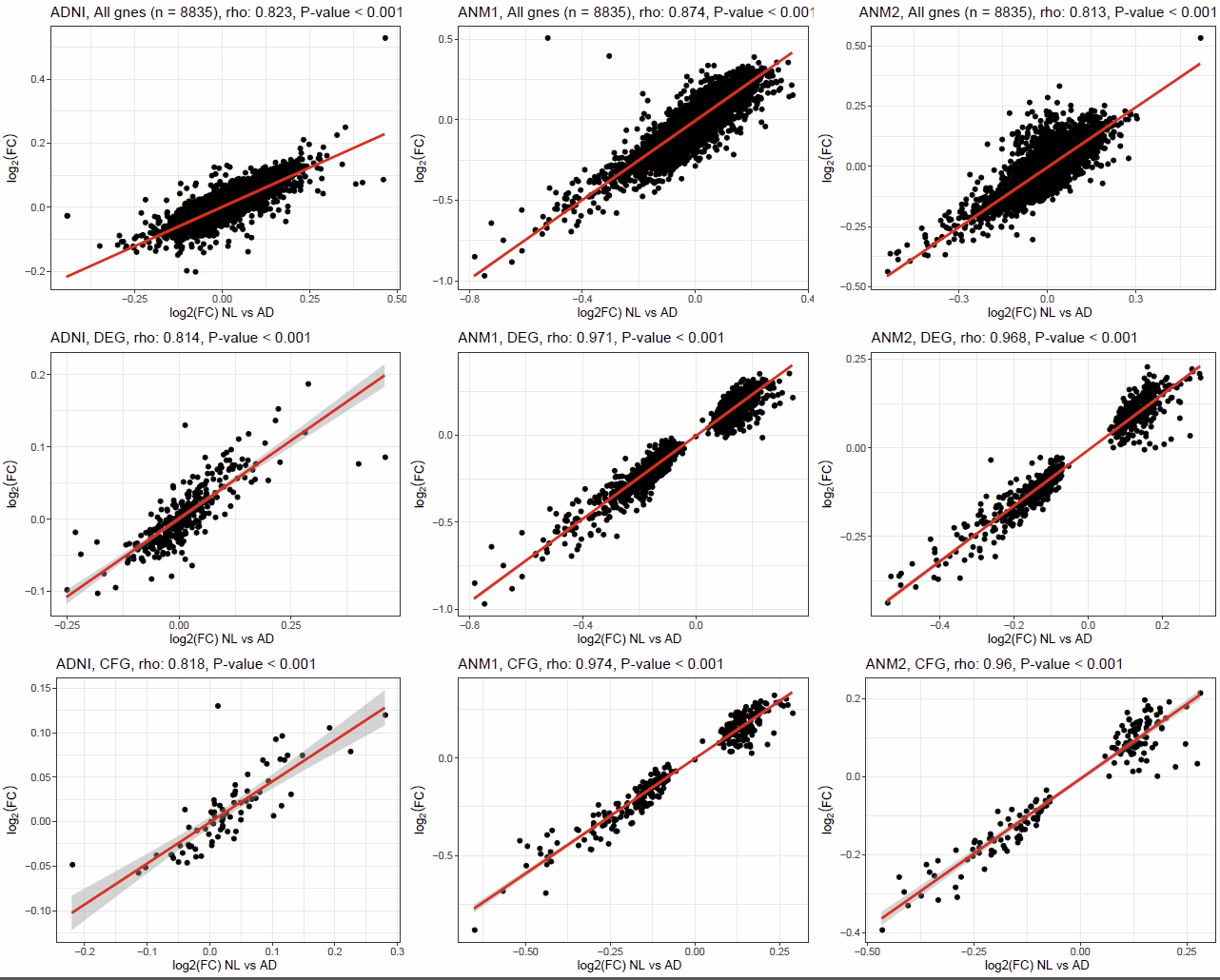


**Supplement Figure 5.** Comparison of the log_2_(FC) signatures between CN and AD samples among different meta-analysis methods.


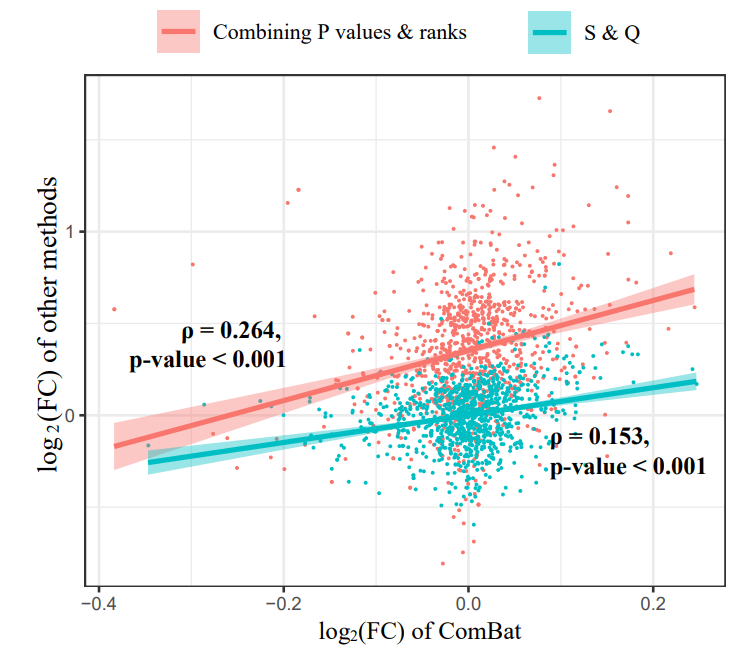


**Supplement Figure 6.** DEGs Venn diagram and P-value for common genes among different meta-analysis methods.


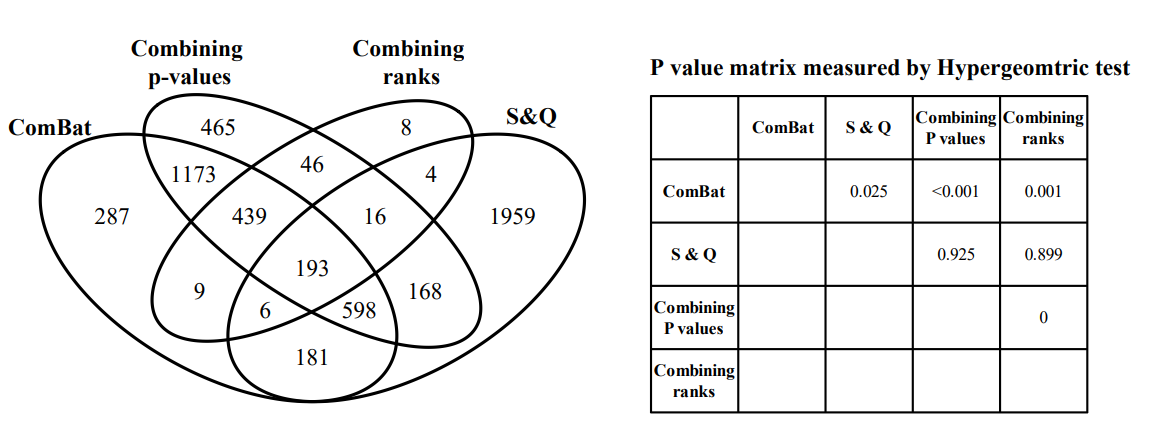


**Supplement Figure 7.** Comparison of performance for classifying AD samples among different meta-analysis methods.


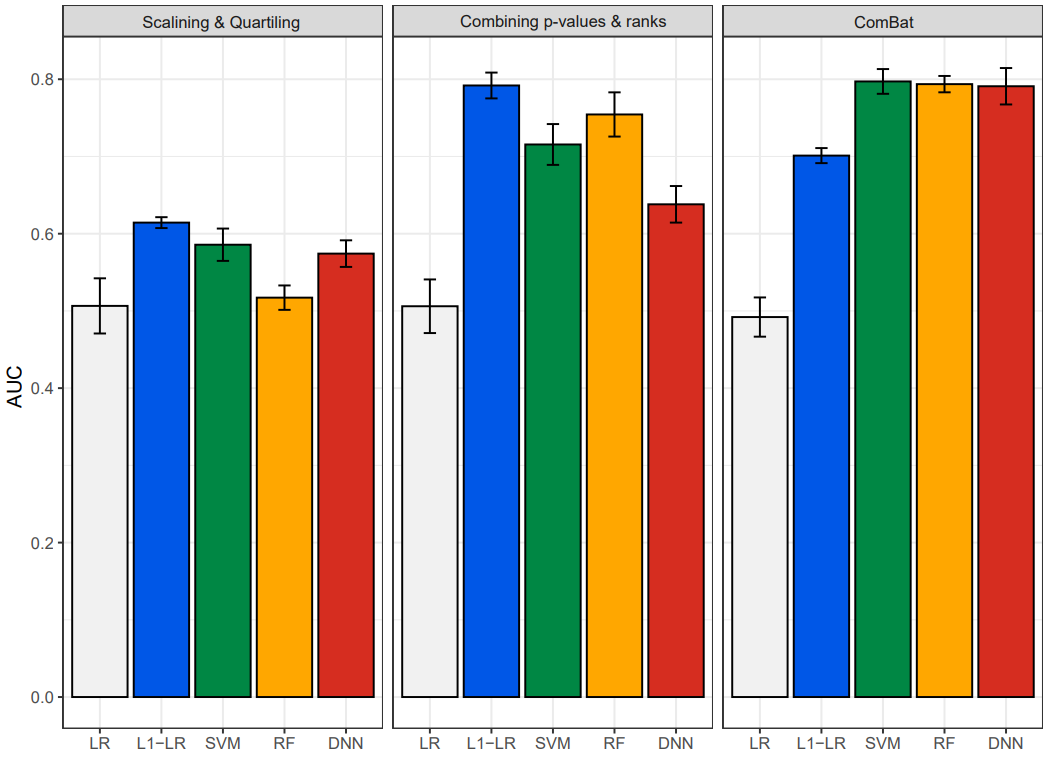


**Related works**

In Table 1, we list previously published blood expression-based studies for the identification of patients with AD, especially focusing on machine learning (ML)-based studies. Booij et al. ^1^ published a study consisting of 320 subjects with microarray-based gene expression data. The variable selection was performed via the Jack-knife and a double cross validation (CV) method minimizing overfitting, yielding a set of 1,239 significant genes. Using 1239 genes, partial least square regression (PLSR) was employed to distinguish AD patients from controls, yielding an accuracy of 0.87 and AUC of 0.94 for the AD classification.

Lunnon et al. ^2^ published an article including 356 samples consisting of CN, MCI, and AD cases with blood gene expression values from ANM. They initially performed a t-test using training samples, which generated 203 probes with q-values (FDR adjusted) < 0.01. Out of the 203 probes, the 50 highly ranked probes were finally selected using the Meng score ^3^. Then, these 50 probes were used as feature variables and the RF model was used as the classifying method, yielding correctly classified 39 / 52 samples with an accuracy of 0.75 ^2^.

Sood et al. ^4^ reported that aging-related genes can serve for discriminating AD from CN. They identified the aging-related genes in 30 muscle samples of 15 young (aged 19 – 28 years) and 15 elderly (aged 59 – 77) individuals without any chronic diseases. By means of an empirical Bayesian statistic and a leave-one-out CV (LOOCV) process, a 150-RNA probe set list was compiled. These 150 probes were used as input of a k-nearest neighbor (kNN) classification method to discriminate AD cases, yielding AUCs of 0.73 and 0.66 in ANM1 and ANM2 using LOOCV, respectively.

Voyle et al. ^5^ analyzed 748 subjects: 614 from ANM and 134 from the Dementia Case Registry (DCR). They used the recursive feature elimination (RFE) and constructed a tolerance set that selected a smaller set of variables maintaining model accuracy. At first, 261 variables were selected by the top 5% of bootstrapped variable importance calculations, and the RFE reduced them to 13 probes (12 genes). Then, the 13 probes were used as the input of RF to classify AD cases form CN. As a result, the RF model correctly discriminated 155 of 236 (AD: 118, CN: 118) with an accuracy of 0.657, and the AUC in this model was 0.724.

Li et al. ^6^ presented in 2017 research consisting of blood gene expression data from GSE63060 (ANM1) and GSE63061 (ANM2). Feature selection was performed by a Ref-REO method, which selected gene pairs with similar gene expression alteration patterns based on leukocyte subtypes, yielding 1,145 and 1,249 reverse gene pairs in ANM1 and ANM2 datasets, respectively. Then, using these features, AUC values of 0.733 and 0.775 were obtained when ANM2 and ANM1 datasets were used as test sets, respectively.

Li et al. ^7^ performed a study involving blood gene expression data from ANM (GSE63060 and GSE63061). For GSE63061, individuals with Western European and Caucasian ethnicity were selected. They used the least absolute shrinkage and selection operator (LASSO) regression that shrinks coefficients with sparsity to curate final variables, resulting in 6 features (Full6set) being selected. They used 6 genes for three ML methods, including SVM, logistic ridge regression (RR), and RF ^7^, yielding mean AUCs of 0.864 and 0.866 for testing ANM1 and ANM2, respectively.

Although these studies have been performed, it is unclear whether the classifiers trained using one AD gene expression data set can be applied to other AD data sets. Thus, in this study, we systematically evaluated five feature selection methods and classifiers for distinguishing individuals with AD from healthy controls (CNs) using three independent blood gene expression datasets.

**Methods**

The ADNI consisted of participants recruited at 57 sites in the US and Canada, funded as a private-public partnership ^8^. The ANM consortium is a large cross-European AD biomarker study and a follow-on DCR cohort in London ^9^. In both ADNI and ANM, AD was diagnosed using the NINCDS-ADRDA criteria for possible or probable AD ^10^.

We employed three large-scale blood gene expression datasets: ANM1 (GEO:GSE63060), ANM2 (GEO:GSE63061), and ADNI (adni.loni.usc.edu, last downloaded 2018/8/31). The overall framework of our study is illustrated in Figure 1. The performance was evaluated via internal validation (five-fold CV within each dataset) and external validation (training and test sets from different datasets).

The extraction of differentially expressed genes (DEGs), logistic regression (LR), L1-LR, support vector machine (SVM), and random forest (RF) were implemented using R (version 3.4.0) and Bioconductor (release 3.8) ^11, 12^. Variational autoencoder (VAE) and deep neural network (DNN) were implemented with C++ based TensorFlow with a Python interface ^13^.

***Preprocessing of data***

Gene expression data were produced using the Affymetrix Human Genome U 219 array for the first case-control study (ADNI), Illumina Human HT-12 v.3 Expression BeadChips for the second case-control study (ANM1), and Illumina Human HT-12 v4 Expression BeadChips for the third case-control study (ANM2).

We performed three steps of data processing. The first step involved the selection of samples and probes to be analyzed. For ADNI, we selected high-quality RNA samples with RIN ≥ 6.9, as the previous study performed ^14^, and for ANM1 and ANM2, we did not exclude any samples. The ADNI, ANM1, and ANM2 datasets consisted of 49,386, 30,063, and 29,485 probes, respectively. The median RNA expression value of ADNI, ANM1, and ANM2 were 3.897, 7.584 and 6.154, respectively, indicating that ADNI could be influenced by background noise due to relatively low gene expression intensities. To reduce the background noise of ANDI, we excluded probes with an intensity value ≤ the median of all gene expression values in 100 or more samples, as performed in the previous study ^15^. If there were multiple probes annotated in one gene, then the median value of those was selected, yielding 11,276, 21,698, and 22,338 unique probes in ADNI, ANM1, and ANM2, respectively. We selected only probes that were present in all three datasets, and 8,835 final probes were left for analysis.

The second step was normalization within each dataset and renormalization between datasets. The probe set level intensities of all three datasets were normalized by the Robust Multi-Array Analysis (RMA) method ^16^. Although each dataset was normalized, a variance or batch effect among different datasets remained. Therefore, we renormalized all three datasets to reduce the batch effect among different datasets using ComBat from the sva package in R ^17^.

The third step was selecting DEGs of patients with AD. We extracted DEGs between the control and AD in the ADNI by the significance analysis of microarrays (SAM), which is a method for identifying genes on a microarray with statistically significant changes in expression ^18^. It was developed in the context of an actual biological experiment. Differentially expressed genes of AD in ANM1 and ANM2 were curated via “lmFit” and “eBayes” functions in the limma package, which is based on a linear regression method ^19^. We set the cutoffs of FDR of the SAM and limma to 0.05 and 0.01, respectively.

***Feature selection (VAE)***

We used VAE to extract a representation from a set of input features thereby reducing the dimensions of the data ^20^. An autoencoder is a type of neural network used to learn efficient and representative information in an unsupervised manner. Specifically, VAE not only adopts the autoencoder architecture, but also assumes that the distribution of encoding features are similar to that of original features ^20^. The objective of VAE are as follows:

$$\mathcal{L}\left( \phi, \theta, x \right)= -E_{z\sim q_{\phi}\left( z|x \right)}\left( \log_{p_{\theta}} \left( x|z \right) \right)+D_{\mathrm{KL}}\left( q_{\phi}\left( z|x \right)||p_{\phi}\left( z \right) \right)$$

where DKL indicates the Kullback-Leibler divergence, and we assume that *q_ϕ_(z|x)*, the distribution of the sampling function, and *p_θ_(z)*, the distribution of encoding features, are the Gaussian and the standard normal distribution, respectively. The framework of VAE is concisely described in Figure 1 and precisely in Supplementary Figure S1A. The structure of VAE was as follows: input features x are expression values of n genes and they are multiplexed by weights, and passed to activation functions, which are elu ^21^, softplus ^22^, and hyperbolic tangent ^23^ in encoding layer, sampling function, and decoding layer, respectively. The reduced features *z* and parameters *θ* and *ϕ* are obtained by maximizing the object function L, and the number of reduced dimensions was set to be similar to the number of selected genes by the CFG score. In a simulation, the distribution of input and encoding features of ADNI and the correlation between the input and reconstructed features are described in Supplementary Figure S1 BD. Furthermore, we tuned a list of trained weights using prior information (sample label: AD or CN) of the training dataset to reduce information loss of shrunken dimension (Supplementary Figure S1A). Note that all processes of VAE were performed on a training dataset. We optimized our VAE model utilizing the Adagrad optimizer ^24^, and the number of iterations and learning rate were set as 3000 and 0.001, respectively.

***Feature selection (CFG)***

The CFG approach is a translational methodology that integrates multiple lines of external evidences from human and animal model studies ^25, 26^. There have been several studies using various CFG scoring methods, from which we selected two representative methods ^27, 28^. The first method ^27^ was to score genes validated by multi-genomic and experimental studies using the following five criteria:

1. genes validated by GWAS in the International Genomics of Alzheimer’s Project with at least one locus being significantly associated with AD ^29^
2. genes associated with SNP genotypes (eQTL) associated with AD risk ^29, 30^
3. genes physically interacting with any AD core genes analyzed by the previous study ^27^
4. genes correlating with AD pathology in AD mice (P < 0.05) at the expression level ^31^
5. genes with early differential expression in AD mice (P < 0.05) ^31^

We assigned each DEG one point if the DEG satisfied one of the above five criteria, yielding a score ranging from 0 to 5 points by using a publicly accessible database at http://alzdata.org ^27^. The second method is the database-based CFG scoring method that uses external lines of evidence ^28^. We scored a gene as one point if the gene was included in the AD-related genes extracted from two databases, including Alzgene and DigSee ^32, 33^, yielding a score ranging from 0 to 2. Bertram et al. constructed a publicly available, continuously updated database (AlzGene, http://www.alzgene.org) by performing systematic meta-analysis for each polymorphism with available genotype data in at least three case-control subjects ^32^. The DigSee extracted gene-disease relationships by incorporating the text-mining method and the machine learning technique and included 4,494 disease types and 13,054 genes ^33^. From the AlzGene and DigSee, we obtained a list of 680 and 1602 AD-related genes, respectively. Combining these two CFG scoring methods, we annotated all DEGs with numeric points ranging from 0 to 7. We defined DEGs with 3 or more points as highly informative AD genes. Lists of DEGs with CFG score (ADNI, ANM1, and ANM2) are presented in Supplementary Tables S2–S4.

***Meta-analysis methods***

To integrate the ADNI, ANM1, and ANM2 datasets, we used four approaches as described in the main manuscript. Methods by Sharifi et al.^10^ and Farhadian et al.^12^ were performed by using the MetaDE package ^13^. We used a “MetaDE.match” function with interquartile range (IQR) setting to select the probe with the largest IQR value among all possible probes for a gene. In addition, we removed approximately 10% probes with low average expression values across the majority of studies, and approximately 10% of probes with minimal amounts of variation.

As Mohammadi-Dehcheshmeh et al.^11^ proposed, we used the Z-standardization by subtracting the mean and dividing by the standard deviation. Afterwards, the scaling and quartiling approaches were applied. In the scaling approach, the sets of the expression values for the samples were weighted by a constant defined by median mean/median median ratio. In the quartiling approach, a common target distribution was calculated by the empirical distribution of gene expression matrix, and used to curate normalized sets of expression values for each sample. Pseudocode for these processes was presented in the study by Mohammadi-Dehcheshmeh et al ^11^.

We named the study by Sharifi et al.^10^ and the study by Farhadian et al.^12^ as “Combining ranks” and “Combining P values”, respectively, based on the DEGs integration methods. In addition, we named the ComBat approach and the study by Mohammadi-Dehcheshmeh et al ^11^ as “ComBat” and “S&Q (Scaling & Quartiling)”, respectively, based on the batch removal method.

***Performance assessment***

Performance was evaluated via internal (five-fold CV within each dataset) and external validation (training and test sets from different datasets). In the external validation, when ADNI data was used as a training set, the other two datasets, ANM1 and ANM2, were used as test sets. Similarly, when ANM1 (ANM2) was used a training set, the other two datasets ADNI and ANM2 (ADNI and ANM1), were used as test sets. In total, six external validations are performed.

An Area under the Receiver Operator Characteristic Curve (AUC) between true positives and false positives was used to assess performance of AD classification models [15, 18-21]. For comparison of results, we also utilized the AUC to quantify the respective classifying power of each model. Owing to the fact that VAE and DNN models generated different outputs depending on initialized values of parameters, performance was measured as the average value of 10 outputs from 10 different operations.

In the internal validation, to obtain statistical significance of difference of performances, we applied a *t*-test using five AUC values obtained from five-fold CV sets (Supplementary Table S5). For the comparison of the five feature selection methods, for each feature selection method, we curated the 25 AUC values from the five cross-validation sets and five classifiers. Then, the *t*-test was used for the comparison of one feature selection method and the other four features selection methods (25 AUC values of one feature selection method *vs* 100 AUC values of the other four feature selection methods).

In the external validation, because only one AUC was resulted, we used a permutation-based approach proposed by Venkatraman et al.^38^ with the setting number of iterations as 200. We compared the five AUC values obtained from five classifiers in each feature selection method (Supplementary Table S6). To compare the performances across the five feature selection methods, we set the AUC obtained from the DEG as the reference, and compared other four AUC values from other four feature selection methods with the reference (Table S6). We only checked significant results in Supplementary Table S6.

For the six comparisons in the external validation (Figure 3), we selected the feature selection method with the best performance, yielding five AUC values from the five classifiers. We compared the five AUC values with other 20 AUC values via a *t*-test.

***Adjustment of age and gender in the batch normalization***

We adjusted gene expression values for confounding factors in the step of batch removal as other studies performed ^39^. The adjustment was conducted using a “ComBat” function with the setting “mod” as “matrix( ~ age + gender)” in the sva package. After removing batch effects of three datasets (ADNI, ANM1, and ANM2), and adjusting age and gender for datasets, we established 25 AD prediction models using five feature lists (DEG, VAE, TF, Hub genes, and CFG) and five classifiers (LR, L1-LR, SVM, RF, and DNN). We made these 25 prediction models for each of the following cases as the same as our previous external validation (training & test sets): ADNI & ANM1, ADNI & ANM2, ANM1 & ADNI, ANM1 & ANM2, ANM2 & ADNI, and ANM2 & ANM1.

**Reference**

1. Booij BB*, et al.* A gene expression pattern in blood for the early detection of Alzheimer's disease. *J Alzheimers Dis* **23**, 109-119 (2011).

2. Lunnon K*, et al.* A blood gene expression marker of early Alzheimer's disease. *J Alzheimers Dis* **33**, 737-753 (2013).

3. Meng YA., Yu Y., Cupples LA., Farrer LA & Lunetta KL. Performance of random forest when SNPs are in linkage disequilibrium. *BMC Bioinformatics* **10**, 78 (2009).

4. Sood S*, et al.* A novel multi-tissue RNA diagnostic of healthy ageing relates to cognitive health status. *Genome Biol* **16**, 185 (2015).

5. Voyle N*, et al.* A pathway based classification method for analyzing gene expression for Alzheimer’s disease diagnosis. *J Alzheimers Dis* **49**, 659-669 (2016).

6. Li H*, et al.* Identification of molecular alterations in leukocytes from gene expression profiles of peripheral whole blood of Alzheimer’s disease. *Sci Rep* **7**, 14027 (2017).

7. Li X*, et al.* Systematic analysis and biomarker study for Alzheimer’s disease. *Sci Rep* **8**, 17394 (2018).

8. Mueller SG*, et al.* Ways toward an early diagnosis in Alzheimer’s disease: the Alzheimer’s Disease Neuroimaging Initiative (ADNI). *Alzheimers Dement* **1**, 55-66 (2005).

9. Lovestone S*, et al.* AddNeuroMed—the European collaboration for the discovery of novel biomarkers for Alzheimer's disease. *Ann N Y Acad Sci* **1180**, 36-46 (2009).

10. McKhann G*, et al.* Clinical diagnosis of Alzheimer's disease: Report of the NINCDS‐ADRDA Work Group* under the auspices of Department of Health and Human Services Task Force on Alzheimer's Disease. *Neurology* **34**, 939-939 (1984).

11. Team RC. R: A language and environment for statistical computing. (2013).

12. Gentleman RC*, et al.* Bioconductor: open software development for computational biology and bioinformatics. *Genome Biol* **5**, R80 (2004).

13. Abadi M*, et al.* Tensorflow: A system for large-scale machine learning. In: *12th {USENIX} Symposium on Operating Systems Design and Implementation ({OSDI} 16)* (ed^(eds) (2016).

14. Hokama M*, et al.* Altered expression of diabetes-related genes in Alzheimer's disease brains: the Hisayama study. *Cereb Cortex* **24**, 2476-2488 (2013).

15. Antonell A*, et al.* A preliminary study of the whole-genome expression profile of sporadic and monogenic early-onset Alzheimer's disease. *Neurobiol Aging* **34**, 1772-1778 (2013).

16. Irizarry RA*, et al.* Exploration, normalization, and summaries of high density oligonucleotide array probe level data. *Biostatistics* **4**, 249-264 (2003).

17. Leek JT., Johnson WE., Parker HS., Jaffe AE & Storey JD. The sva package for removing batch effects and other unwanted variation in high-throughput experiments. *Bioinformatics* **28**, 882-883 (2012).

18. Tusher VG., Tibshirani R & Chu G. Significance analysis of microarrays applied to the ionizing radiation response. *Proc Natl Acad Sci U S A* **98**, 5116-5121 (2001).

19. Smyth G. Linear models and empirical bayes methods for assessing differential expression in microarray experiments. *Stat Appl Genet Mol Biol* **3**, Article 3 (2004).

20. Kingma DP & Welling M. Auto-encoding variational bayes. *arXiv preprint arXiv:13126114*, (2013).

21. Clevert D-A., Unterthiner T & Hochreiter S. Fast and accurate deep network learning by exponential linear units (elus). *arXiv preprint arXiv:151107289*, (2015).

22. Nair V & Hinton GE. Rectified linear units improve restricted boltzmann machines. In: *Proceedings of the 27th international conference on machine learning (ICML-10)* (ed^(eds) (2010).

23. Karlik B & Olgac AV. Performance analysis of various activation functions in generalized MLP architectures of neural networks. *International Journal of Artificial Intelligence and Expert Systems (IJAE)* **1**, 111-122 (2011).

24. Duchi J., Hazan E & Singer Y. Adaptive subgradient methods for online learning and stochastic optimization. *J Mach Learn Res* **12**, 2121-2159 (2011).

25. Niculescu AB & Le-Niculescu H. Convergent Functional Genomics: what we have learned and can learn about genes, pathways, and mechanisms. *Neuropsychopharmacology* **35**, 355 (2010).

26. NICULESCU III AB*, et al.* Identifying a series of candidate genes for mania and psychosis: a convergent functional genomics approach. *Physiol Genomics* **4**, 83-91 (2000).

27. Xu M*, et al.* A systematic integrated analysis of brain expression profiles reveals YAP1 and other prioritized hub genes as important upstream regulators in Alzheimer's disease. *Alzheimers Dement* **14**, 215-229 (2018).

28. Breen MS*, et al.* Candidate gene networks and blood biomarkers of methamphetamine-associated psychosis: an integrative RNA-sequencing report. *Transl Psychiatry* **6**, e802 (2016).

29. Lambert J-C*, et al.* Meta-analysis of 74,046 individuals identifies 11 new susceptibility loci for Alzheimer's disease. *Nat Genet* **45**, 1452 (2013).

30. Colantuoni C*, et al.* Temporal dynamics and genetic control of transcription in the human prefrontal cortex. *Nature* **478**, 519 (2011).

31. Matarin M*, et al.* A genome-wide gene-expression analysis and database in transgenic mice during development of amyloid or tau pathology. *Cell Rep* **10**, 633-644 (2015).

32. Bertram L., McQueen MB., Mullin K., Blacker D & Tanzi RE. Systematic meta-analyses of Alzheimer disease genetic association studies: the AlzGene database. *Nat Genet* **39**, 17-23 (2007).

33. Kim J., Kim JJ & Lee H. An analysis of disease-gene relationship from Medline abstracts by DigSee. *Sci Rep* **7**, 40154 (2017).

34. Sharifi S*, et al.* Integration of machine learning and meta-analysis identifies the transcriptomic bio-signature of mastitis disease in cattle. *PLoS One* **13**, e0191227 (2018).

35. Farhadian M., Rafat SA., Hasanpur K., Ebrahimi M & Ebrahimie E. Cross-Species Meta-Analysis of Transcriptomic Data in Combination With Supervised Machine Learning Models Identifies the Common Gene Signature of Lactation Process. *Frontiers in genetics* **9**, 235 (2018).

36. Wang X., Li J., Tseng GC & Wang MX. Package ‘MetaDE’. (2012).

37. Mohammadi-Dehcheshmeh M*, et al.* Unified Transcriptomic Signature of Arbuscular Mycorrhiza Colonization in Roots of Medicago truncatula by Integration of Machine Learning, Promoter Analysis, and Direct Merging Meta-Analysis. *Frontiers in plant science* **9**, 1550 (2018).

38. Venkatraman ES & Begg CB. A distribution-free procedure for comparing receiver operating characteristic curves from a paired experiment. *Biometrika* **83**, 835-848 (1996).

39. Gandal MJ*, et al.* Shared molecular neuropathology across major psychiatric disorders parallels polygenic overlap. *Science* **359**, 693-697 (2018).
